# Efficacy and safety of butylphthalide as adjunctive therapy for vascular dementia: A protocol for systematic review and meta-analysis

**DOI:** 10.1097/MD.0000000000023236

**Published:** 2020-11-13

**Authors:** Ling Zhou, Rong Yang, Feiyue Wu

**Affiliations:** Zhoushan Hospital, Zhejiang University School of Medicine, Zhejiang, China.

**Keywords:** adjunctive therapy, butylphthalide, complementary medicine, evidence-based medicine, vascular dementia

## Abstract

**Background:**

Butylphthalide is widely used for the adjunctive treatment of vascular dementia; however, the clinical evidences are not well synthesized yet.

**Methods:**

We proposed a systematic review and meta-analysis to evaluate the efficacy and safety of butylphthalide as adjunctive therapy for vascular dementia. Seven electronic databases (China National Knowledge Infrastructure, Wanfang database, Chongqing VIP database, China Biomedical Literature Database, Pubmed, EMBASE and Cochrane library) will be searched for eligible randomized controlled trials (RCTs). Required data of included studies will be collected. Quality of studies will be evaluated using Cochrane risk of bias assessment tool. Data synthesis will be performed using Review Manager software. Subgroup analysis and sensitivity analysis will also be carried.

**Results:**

Synthesis results of current available RCTs regarding the efficacy and safety of butylphthalide for the treatment vascular dementia will be provided by this systematic review and meta-analysis.

**Conclusion:**

This systematic review and meta-analysis will provide high level evidence of butylphthalide clinical application.

**Registration:**

PROSPERO CRD42020168947

## 1 Introduction

Vascular dementia is a kind of brain circulation dysfunction and brain damage syndrome because of cerebrovascular factors, resulting clinical manifestations of cognitive disorder, cerebrovascular pathologies and progressive memory loss. It leads to severe impact on quality of life and health.^[1,2]^ It is the second most common type of dementia after Alzheimer disease, and it has attracted increasing attention.^[3,4]^ But the molecular and cellular mechanisms of vascular dementia remain poorly understood.^[5–7]^ Pharmacological therapy is a common option for treatment of vascular dementia and many drugs have been used in clinical practice; however, no drug has a specific indication of vascular dementia.^[5]^ Drug therapy will be better informed once additional information of pathological, biomarker and genetic studies are available.^[4]^ So physicians have to turn their attention to complementary therapy and adjunctive therapy.^[8,9]^

Butylphthalide was first extracted from the celery seed and was discovered within the last few decades.^[10]^ The main pharmacologic effects of butylphthalide include antiplatelet aggregation effect, mitochondria protect effect, antiapoptotic effect, and so on.^[11–14]^ Based on the therapeutic property of this compound, butylphthalide was artificially synthesized later in China and approved by the China Food and Drug Administration for ischemic stroke and neuro impairment induced by ischemic stroke.^[10]^ Due to its promising pharmacological effect and good safety profile, butylphthalide is widely used as an adjunctive therapy for ischemic stroke.^[15]^ It is also used for neurodegenerative diseases such as Alzheimer disease and Parkinson disease.^[10]^ Butylphthalide is believed to improve vascular dementia management through its antioxidant, anti-apoptotic effects and through reduction of A*β* deposits.^[11]^ It is also recognized as hopeful adjunctive therapy for vascular dementia. Recently, many experimental and clinical studies have accumulated evidences about the effect of butylphthalide for the treatment of vascular dementia.^[16–18]^

However, there is no systematic review and meta-analysis which evaluates the clinical efficacy and safety of butylphthalide as adjunctive therapy for vascular dementia. So we draft this protocol and prepare to synthesis the current clinical evidence regarding the efficacy and safety of butylphthalide for vascular dementia using a systematic review and meta-analysis method.

## 2 Methods

This systematic review and meta-analysis protocol was drafted following the Preferred Reporting Items of Systemic Review and Meta-Analysis Protocol (PRISMA-P) statement.^[19]^ This protocol was registered in PROSPERO with the registration number CRD42020168947. Ethical approval was not required, because only published studies would be involved in this systematic review and meta-analysis.

### 2.1 Eligibility criteria

#### 2.1.1 Study design

Only randomized controlled trial (RCTs) will be included. Blinding is not necessary but will be a factor to evaluate quality.

#### 2.1.2 Participants

Adults with a diagnosis of vascular dementia will be included. There is no restriction regarding the diagnostic criteria. Patients at end-stage of other disease and have an expected life <6 months will be excluded.

#### 2.1.3 Intervention

Vascular dementia patients treated with butylphthalide combined conventional therapy will be included. There is no restriction regarding the formulation or dose of butylphthalide.

#### 2.1.4 Comparator

Vascular dementia patients treated with any recognized conventional therapy will be included. Conventional therapy can be indicated as pharmacological therapy, rehabilitation therapy, complementary therapy (such as acupuncture), and placebo therapy.

#### 2.1.5 Language

There is no restriction of publication language.

### 2.2 Information source

The following electronic databases will be considered as information sources and searched: China National Knowledge Infrastructure (CNKI), Wanfang database, Chongqing VIP database, China Biomedical Literature Database (CBM), Pubmed, EMBASE, and Cochrane library.

### 2.3 Search strategy

A combination of following key words will be used to run the electronic search of databases: butylphthalide, N-butylphthalide, 3-n-butylphthalide, L-3-n-butylphthalide, DL-3-n-butylphthalide, vascular dementia, VD, VaD, vascular cognitive impairment. The search strategy will be modified to adapt specific database. Manual search will be carried out for relevant studies (such as reference of searched studies). Studies will be restricted to those which published before April 1^st^, 2020. The literature search will be run by two reviewers (LZ and RY) independently, and any discrepancy will be resolved with a third reviewer (FW). The literature search will be re-run before final data synthesis.

### 2.4 Study records

#### 2.4.1 Study selection

The retrieved studies will be managed using a reference software, NoteExpress. Two reviewers (LZ and RY) will select the studies according to the eligibility criteria independently. Reasons of exclusion for each study will be recorded. The results will be cross-checked, and any discrepancies will be resolved with a third reviewer (FW).

#### 2.4.2 Data collection

The following items of included studies will be collected: first author and published year, study duration and religion, participant information of intervention group and comparator group (numbers, average age and age range, sex, severity of illness), butylphthalide dose, formulation and treatment length of intervention group, combined therapy of intervention group, treatment of comparator group, outcomes and measure times, adverse events, and occurrence time. If required items are not shown in the published literature, a reviewer (FW) will contact the corresponding author of original study for detailed information by e-mail. The extracted information will be managed using an Excel sheet. The data collection process will be carried out by 2 reviewers (LZ and RY) independently, and any discrepancy will be resolved with a third reviewer (FW).

### 2.5 Outcomes

#### 2.5.1 Main outcome

The following outcomes measure at the last available follow-up of original study will sought as main outcome of this systematic review and meta-analysis: Mini-mental state examination (MMSE) score; Activities of daily living (ADL) score; Adverse event rate.

#### 2.5.2 Secondary outcome

Barthel index. The secondary outcome will also use the last available follow-up of original study.

### 2.6 Risk of bias in individual studies

Risk of bias in individual studies will be evaluated by 2 reviewers (LZ and RY) using the Cochrane risk of bias assessment tool independently.^[20]^ The following characteristics will be evaluated for each included study: random of sequence generation, allocation concealment, blinding of participants and personnel, blinding of outcome assessment, incomplete outcome data, selective reporting. Each characteristic will be graded to “High risk,” “Low risk,” or “Unclear.” The final results will be cross-checked and any discrepancies will be resolved with a third reviewer (FW).

### 2.7. Data synthesis

2.7

The retrieved data will be reviewed before final synthesis. If included studies for main outcome are <5, only qualitative description will be carried. Data synthesis will be made using Review Manager software (version 5.3, The Cochrane collaboration 2014). *I*^2^ test will be used to assess the heterogeneity among included studies. Mantel-Haenszel fixed-effect model will be used of *I*^2^ <50%, otherwise a random effect model will be employed. Mean difference and 95% confidential interval (CI) will be calculated for continuous results, and odds ratio and 95% CI will be used for categorical result synthesis.

If included studies are sufficient, subgroup analysis will be carried out based on: dose and treatment length of butylphthalide; type of combined therapy; severity of illness.

Sensitivity analysis will also be carried out by comparing the synthesis results using different models (random-effect model vs fixed-effect model). Publication bias will be assessed using funnel plot if eligible studies are >10.

### 2.8 Summary and dissemination plan

The Grading of Recommendation, Assessment, Development, and Evaluation approach will be used for the summary of this systematic review and meta-analysis.^[21]^ Discussion will be made about the strength, implication, and limitation of this study. The final systematic review and meta-analysis will be drafted following the Preferred Reporting Items of Systemic Review and Meta-Analysis (PRISMA) statement and submitted to international peer-reviewed journal.^[22]^ The results will also be presented in conferences.

## 3 Discussion

This systematic review and meta-analysis protocol ensures the quality and transparency of final result. It will be the first systematic review and meta-analysis about the efficacy of butylphthalide as adjunctive therapy for the treatment of vascular dementia. It will provide high-level evidence of butylphthalide application. The strength and limitation of current RCTs about the efficacy of butylphthalide will also be evaluated; thus, this research will be helpful to further clinical study design of butylphthalide.

## Author contributions

Conceptualization, LZ; Funding acquisition, FW; Methodology, LZ, RY and FW; Resources, LZ; Software, RY; Supervision, LZ; Writing – original draft, LZ and RY; Writing – review & editing, LZ, RY and FW.

**Conceptualization:** Ling Zhou.

**Funding acquisition:** Feiyue Wu.

**Methodology:** Ling Zhou, Rong Yang, Feiyue Wu.

**Resources:** Ling Zhou.

**Software:** Rong Yang.

**Supervision:** Ling Zhou.

**Writing – original draft:** Ling Zhou, Rong Yang, Feiyue Wu.

**Writing – review & editing:** Ling Zhou, Rong Yang, Feiyue Wu.
